# Cerebellar Ataxia Followed by Stiff Person Syndrome in a Patient with Anti-GAD Antibodies

**DOI:** 10.1155/2020/8454532

**Published:** 2020-02-08

**Authors:** Sinali O. Seneviratne, Katherine A. Buzzard, Belinda Cruse, Mastura Monif

**Affiliations:** ^1^Curtin University, Kent Street, Bentley, Perth, Western Australia, Australia; ^2^Department of Neurology, Royal Melbourne Hospital, Parkville, Melbourne, Victoria, Australia; ^3^Eastern Health Clinical School, Faculty of Medicine, Nursing and Health Sciences, Monash University, Box Hill Hospital, Box Hill, Melbourne, Victoria, Australia; ^4^Department of Neurology, Alfred Health, Melbourne, Victoria, Australia; ^5^Department of Physiology, University of Melbourne, Melbourne, Victoria, Australia; ^6^Department of Neuroscience, Monash University, Melbourne, Victoria, Australia

## Abstract

Anti-GAD antibody syndrome is a result of the production of antibodies against glutamic acid decarboxylase (GAD), the main enzyme responsible for the production of gamma-aminobutyric acid (GABA). Several neurological manifestations including cerebellar ataxia and stiff person syndrome have been reported in association with anti-GAD antibodies. In this paper, we present a case of a young woman with anti-GAD antibodies who initially presented with cerebellar ataxia followed by stiff person syndrome three and a half years later. Having both cerebellar ataxia and stiff person syndrome is a rare occurrence in anti-GAD antibody syndrome. We emphasise the importance of long-term follow-up of patients with anti-GAD antibody syndrome, as delayed neurological manifestations can occur.

## 1. Introduction

GAD is the main enzyme responsible for the production of GABA, an inhibitory neurotransmitter of the central nervous system (CNS) [1]. Antibodies against GAD have long been associated with the development of type 1 diabetes mellitus. A much rarer association is with the development of neurological syndromes, including cerebellar ataxia, stiff person syndrome, limbic encephalitis and encephalopathy, seizures, eye movement disorders, and Miller Fisher Syndrome [2]. Neurological anti-GAD antibody syndromes have been reported in the context of a paraneoplastic syndrome [2]. Cerebellar ataxia commonly presents as gait ataxia, nystagmus, and dysarthria, whereas stiff person syndrome is characterised by painful muscle spasms, intermittent muscle contractions, and heightened startle response. Both conditions may lead to severe gait impairment. Having both cerebellar ataxia and stiff person syndrome is a rare occurrence of which only a few cases have previously been reported [3, 4]. In this paper, we present a patient who initially presented with cerebellar ataxia, and later developed stiff person syndrome as a manifestation of anti-GAD antibody syndrome.

## 2. Case Report

A 36-year-old woman was admitted to a tertiary hospital for investigation of unexplained weight loss (16 kg over 18 months). She had no relevant past medical history and was not taking any medications. One year prior to admission, she was noted to have an unusual “stiff” upright posture, a wide-based ataxic gait, and experienced frequent “jerking” movements in her sleep. Several months leading up to the admission, she started to experience general fatigue, “dizziness,” and self-reported difficulties with her memory. Several weeks prior to her admission, the patient reported “jerky eye movements”, slurred speech, and unsteadiness. Examination on admission confirmed prominent multidirectional nystagmus, dysarthria, and cerebellar ataxia. Several investigations were undertaken in view of her weight loss and neurological symptoms. Stool microscopy, diabetes screen, coeliac serology, thyroid function test, gastroscopy, colonoscopy, bowel MRI, and tumour markers were all normal. The cerebrospinal fluid analysis showed normal biochemical parameters and white cell count within the normal range. Various immunological investigations including anti-Hu, anti-Ri, anti-Yo, anti-PCA-2, anti-CRMP5, anti-PCA-Tr, anti-Ma/Ta, anti-Amphiphysin, anti-thyroid antibodies, anti-neutrophil cytoplasmic antibodies, and celiac antibody screen were negative. Whipple's PCR was negative in CSF. Serum anti-GAD 65 antibodies were significantly elevated (1091 U/mL normal being <5 U/mL; using the RSR ELISA method). Anti-GAD antibodies were detected in the CSF as well. Given the potential association of anti-GAD antibodies and malignancies, the patient underwent a whole-body PET scan which was normal. A bone marrow aspirate and trephine were similarly unremarkable. The patient did not have an EEG.

The patient was initially treated for anti-GAD antibody associated cerebellar ataxia with three days of intravenous (IV) 1 g methylprednisolone and three days of IV immunoglobulins (IVIG; 2 g/Kg), followed by monthly IVIG treatment and a tapering dose of oral prednisolone. Due to ongoing disabling symptoms, 4 months later, the patient received five alternate day sessions of plasma exchange resulting in symptom stabilization. Eight months after initial admission, the patient continued to demonstrate cerebellar ataxia with prominent, nystagmus, dysarthria, and limb dysmetria. The remainder of her neurological examination was unremarkable.

The decision was made to treat the patient with Rituximab (375 mg/m^2^ weekly for 4 weeks). She remained on a moderate dose of prednisolone 10 mg daily. Attempts to wean the prednisolone dose further resulted in worsening of cerebellar ataxia. Two months after the rituximab induction course was completed, the patient reported subjective improvement in her mobility and balance despite ongoing signs of cerebellar dysfunction.

Approximately 18 months after the diagnosis of anti-GAD antibody-associated cerebellar ataxia, the patient was diagnosed with insulin-dependent diabetes mellitus. She was unable to reduce the prednisolone below 10 mg daily due to worsening symptoms. The patient reported wearing off of the initial benefit seen after Rituximab treatment; hence, the decision was made to repeat the Rituximab treatment (1 g IV). Mycophenolate mofetil was subsequently introduced as a maintenance immunosuppressive treatment (initially 500 mg bd) together with prednisolone 10 mg daily.

When the diagnosis was established, the GAD antibody titre was 1091 U/mL. Two years later, after receiving immunotherapy including rituximab, the titre was still elevated at 1134 U/mL.

Five years after her initial presentation with significant unexplained weight loss, the patient developed muscle cramps and spasms particularly affecting the paraspinal musculature, also involving the neck and jaw. The patient underwent a hyperexcitability neurophysiology study (exteroceptive reflex testing). Following the right medial nerve stimulation, there was evidence of positive exteroceptive reflexes typical of spinal reflex myoclonus supporting the diagnosis of anti-GAD antibody-associated stiff person syndrome (Table 1).

Table 1 shows evidence of exteroceptive reflexes following the right median nerve stimulation typical of reflex myoclonus as characterized in stiff person syndrome. When the median nerve was stimulated, there was a reflex EMG activity initially seen at rectus abdominis at a latency of 50.52–63.54 ms. Following this, there was rostral to caudal recruitment of the paraspinals (PSP) and sternocleidomastoid (SCM) with latencies of 49.48–91.15 ms.

MRI brain with contrast was performed when the patient first presented with neurological symptoms, and then annually. The last MRI brain was performed when the patient presented with symptoms of stiff person syndrome. All scans were normal.

## 3. Discussion

We report a young woman who first presented with unexplained weight loss and cerebellar dysfunction. Subsequently, tests revealed anti-GAD antibodies both in serum and in CSF. She was treated with steroids, intravenous immunoglobulins, rituximab, and mycophenolate mofetil. Five years later, while being treated with steroids (10 mg daily) and mycophenolate mofetil (500 mg twice a day), she developed signs and symptoms consistent with stiff person syndrome. This case report highlights the possibility of sequential neurological syndromes due to anti-GAD antibodies.

Anti-GAD antibody syndromes encompass a range of different neurological presentations. Stiff person syndrome is the most frequent manifestation followed by cerebellar ataxia and other neurological syndromes [2]. More recently, the association with autoimmune encephalitis and drug-refractory epilepsy has been described [5]. Other neurological manifestations associated with anti-GAD antibody syndrome include brainstem dysfunction, oculomotor disorders, and palatal myoclonus [5]. These conditions may present as paraneoplastic syndromes [5]. Autoimmune disorders such as type 1 diabetes, autoimmune thyroiditis, and pernicious anaemia are often associated with anti-GAD antibody syndromes [1].

We note that later in the course, the patient was diagnosed with type-1 diabetes. Though anti-GAD antibody was positive, other diabetes-related autoantibodies such as islet cell cytoplasmic autoantibodies (ICA), insulin autoantibodies (IAA), insulinoma-associated-2 autoantibodies (IA-2A), and Zinc Transporter 8 antibodies (ZnT8) were not tested. After the diagnosis of GAD antibody syndrome, she received pulsed IV methylprednisolone and low-dose-maintenance prednisolone. We believe her type-1 diabetes is an association of GAD antibody syndrome, as this is well described in the literature, rather than a result of steroid therapy.

Neurological syndromes associated with anti-GAD antibodies are defined by their presence in the serum and cerebrospinal fluid. GAD enzyme exists in two isoforms, 65 and 67; GAD 65 is the most immunogenic isoform [5]. Both radioimmunoassay and enzyme-linked immunosorbent assay are techniques used to measure anti-GAD antibodies. Immunohistochemistry displays a characteristic pattern with anti-GAD antibody showing presynaptic staining in rat cerebellar preparations, while radioimmunoassay values ≥2000 U/mL is considered the threshold for indicating positivity in patients with neurological syndromes [2]. The detection of GAD antibodies in the cerebrospinal fluid indicating intrathecal synthesis is a strong indication of a neurological syndrome associated with GAD antibody [5].

Management of this condition involves immunosuppression, treatment of the symptoms, and treatment of underlying autoimmune conditions or cancer. Immunotherapy with intravenous immunoglobulins, steroids, and plasmapheresis has been reported to have variable success rates [5]. The reason for inadequate efficacy is thought to be due to the intracellular location of the antigen. Some case reports suggest efficacy of rituximab in stiff person syndrome [6], although a randomised placebo-controlled study failed to demonstrate any significant benefits [7]. For symptom management of spasms and rigidity, GABAergic medications such as benzodiazepines and baclofen are used.

Although anti-GAD antibody-associated neurological syndromes occur in isolation, there are rare case reports of the combination of stiff person syndrome and cerebellar ataxia similar to our case. Those patients who develop stiff person syndrome and cerebellar ataxia have a higher intrathecal synthesis of the GAD antibody compared with patients who present with isolated stiff person syndrome [8]. In our literature review, we found six studies describing ten patients as summarised in Table 2 [3, 4, 8–11]. Out of ten patients, six were females, while type-1 diabetes mellitus was found to be associated with six cases. Three patients demonstrated signs and symptoms of both stiff person syndrome and cerebellar ataxia at the initial presentation. In others, stiff person syndrome was followed by cerebellar ataxia or vice versa. Variable response to intravenous immunoglobulins and steroids was reported among these cases. A more recent case report indicates good response to plasmapheresis and rituximab [11]. Our patient shares several similarities with these cases reported in the literature (female sex, association with type-1 diabetes mellitus, and sequential presentation of stiff person syndrome preceded by cerebellar ataxia).

Anti-GAD antibody-associated neurological syndromes include limbic encephalitis, epilepsy (particularly temporal lobe epilepsy-TLE), stiff person syndrome, and cerebellar syndrome. Some clinical clues may help to select appropriate candidates for antibody testing. A study based on late-onset TLE of unknown etiology found 21% of patients to be anti-GAD antibody positive. Those patients typically had pharmacoresistant epilepsy accompanied by depression, memory impairment, and other autoimmune disorders [12]. Another study evaluated patients presenting with new-onset epilepsy or established epilepsy of unknown etiology and found 12.5% anti-GAD-antibody-positive subjects. They proposed a clinical score (antibody prevalence in epilepsy score, APE score) to select patients for antibody testing. The clinical features of the score include mental state changes, seizures, neuropsychiatric changes, autonomic dysfunction, viral prodrome, pharmacoresistant seizures, CSF abnormalities, brain MRI changes, and underlying malignancies. They found an APE score of ≥4 which predicted positive antibody results with 82% specificity and sensitivity [13]. Overall, anti-GAD-antibody-associated neurological conditions are typically seen in young or middle-aged females and often associated with other autoimmune disorders and antibodies [14, 15].

The presentation raises the possibility of progressive encephalomyelitis with rigidity and myoclonus (PERM) as a potential differential diagnosis. This condition often presents with muscle stiffness, myoclonus, gait ataxia, behavioural changes, and cognitive decline [16]. PERM is typically associated with glycine receptor antibodies, but a small number of patients have detectable anti-GAD antibodies [16]. The overall clinical picture including normal MRI brain scan in our case is in keeping with the diagnosis of anti-GAD antibody neurological syndrome.

In this case report, we highlight the rare combination of stiff person syndrome and cerebellar ataxia due to anti-GAD antibodies. We emphasise the importance of long-term follow-up of these patients, given the possibility of late development of a different neurological syndrome.

## Figures and Tables

**Table 1:** The evidence of exteroceptive reflexes following the right median nerve stimulation typical of reflex myoclonus as characterised in stiff person syndrome. When the median nerve was stimulated, there was a reflex EMG activity initially seen at rectus abdominis at a latency of 50.52–63.54 ms. Following this, there was rostral to caudal recruitment of the paraspinals (PSP) and sternocleidomastoid (SCM) with latencies of 49.48–91.15 ms.

Nerve/sites	Onsetms						
R median nerve-triggered exteroceptive test							
R SCM	70.83		82.29	57.29	86.98	74.48	72.40
R deltoid	76.56	95.31	68.75	63.02	78.65	72.40	62.50
R C7 PSP	65.63	72.40			88.54	70.83	51.04
R T4 PSP	63.54	61.46	49.48		75.00	69.79	46.88
R T12 PSP	50.52	58.85	54.17	59.38	54.69	58.33	56.77
R rectus abdominus	65.63	58.85		66.15	54.69	70.83	57.29

**Table 2:** Previous studies describing the combination of anti-GAD antibody-associated stiff person syndrome and cerebellar ataxia.

Reference	Age, sex	Presentation	Associated immunological disorders	Immunotherapy	Response to immunotherapy
Giometto et al. [9]	55, female	SPS ≥ CA	DM1, PA	Steroids	Partial response
Kono et al. [4]	46, female	CA ≥ SPS	DM1	NS	—
Kim et al. [3]	40, female	SPS + CA	DM1, HT	Steroids	Good response
Rakocevic et al. [8]	30, male	SPS ≥ CA		IVIG	Partial response
	51, female	CA ≥ SPS	DM1, PA	IVIG, steroids	No response
	72, female	SPS ≥ CA		IVIG, steroids	No response
	50, male	SPS ≥ CA	DM1, HT	NS	—
	63, male	SPS ≥ CA	PA	IVIG	No response
Ances et al. [10]	55, female	SPS + CA	—	IVIG	Good response
Schaefer and Moeller [11]	42, male	SPS + CA ≥ LE	DM1, HT	IVIG and steroids Plasmapheresis and rituximab	No response, good response

SPS = stiff person syndrome; CA = cerebellar ataxia; LE = limbic encephalitis; DM1 = type-1 diabetes mellitus; PA = pernicious anaemia; HT = Hashimoto thyroiditis; IVIG = intravenous immunoglobulin; NS = not specified.
